# Gradient Nanostructures and Machine Learning Synergy for Robust Quantitative Surface‐Enhanced Raman Scattering

**DOI:** 10.1002/advs.202501793

**Published:** 2025-04-25

**Authors:** Xiaoyu Zhao, Yuxia Wang, Yuting Liu, Xinyi Chen, Mingyu Cheng, Yaxin Wang, Jiahong Wen, Renxian Gao, Kun Zhang, Fengyi Zhang, Rufei Cui, Yongjun Zhang, Zengyao Wang, Bin Ai

**Affiliations:** ^1^ College of Materials and Environmental Engineering Hangzhou Dianzi University Hangzhou Zhejiang 310018 P. R. China; ^2^ School of Microelectronics and Communication Engieerimng Chongqing Key Laboratory of Bio‐perception & Intelligent Information Processing Chongqing University Chongqing 400044 P. R. China; ^3^ The College of Electronics and Information Hangzhou Dianzi University Hangzhou 310018 P. R. China; ^4^ Shangyu Institute of Science and Engineering Hangzhou Dianzi University Shaoxing Zhejiang 312000 P. R. China; ^5^ Shandong Second Medical University Weifang Shandong 261053 P. R. China

**Keywords:** Gradient Nanostructures, machine learning, shadow sphere lithography, Surface‐Enhanced Raman Scattering

## Abstract

Surface‐Enhanced Raman Scattering (SERS) holds significant promise for trace‐level molecular detection but faces challenges in achieving reliable quantitative analysis due to signal variability caused by non‐uniform “hot spots” and external factors. To address these limitations, a novel SERS platform based on gradient nanostructures is developed using shadow sphere lithography, enabling the acquisition of diverse spectral features from a single analyte concentration under identical conditions. The gradient design minimizes fabrication variability and enhances spectral diversity, while the machine learning (ML) model trained on the multi‐spectral dataset significantly outperformed traditional single‐spectrum approaches, with the test Mean Squared Error (MSE) reduced by 84.8% and the coefficient of determination (*R* ^2^) improved by 61.2%. This strategy captures subtle spectral variations, improving the precision, robustness, and reproducibility of SERS‐based quantification, paving the way for its reliable application in real‐world scenarios.

## Graphical abstract

A novel SERS platform using gradient nanostructures and machine learning is presented. Gradient nanostructures minimize fabrication variability and enhance spectral diversity. The ML model significantly improves quantification precision, robustness and reproducibility, reducing test MSE by 84.8% and increasing R^2^ by 61.2%, enabling reliable real‐world applications.

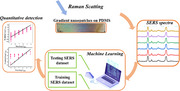

## 1 Introduction

Surface‐Enhanced Raman Scattering (SERS) has emerged as a transformative analytical technique, offering unparalleled sensitivity for detecting low‐concentration analytes. By leveraging the localized surface plasmon resonance (LSPR) of metallic nanostructures, SERS enhances Raman signals by several orders of magnitude, enabling trace‐level detection of molecules. This capability has catalyzed significant advancements in diverse fields such as chemical sensing,^[^ ^1^ ^]^ biological diagnostics,^[^ ^2^ ^]^ environmental monitoring,^[^ ^3^ ^]^ and forensic science.^[^ ^4^ ^]^ However, achieving reliable and consistent quantitative analysis with SERS remains a formidable challenge due to the inherent variability of SERS signals, which are highly sensitive to experimental conditions and subtle environmental changes.^[^ ^4^ ^]^The primary source of this variability lies in the inhomogeneous distribution of “hot spots”—regions of intense electromagnetic field enhancement—on nanostructured substrates. These hot spots are often non‐uniform and difficult to reproduce, leading to signal fluctuations that compromise both accuracy and reproducibility.^[^ ^5^, ^6^ ^]^ Additionally, external factors such as laser power instability, substrate degradation, and variations in the local environment further exacerbate inconsistencies in SERS measurements.^[^ ^7^ ^]^ Addressing these issues is critical for transitioning SERS from a qualitative tool to a robust quantitative method suitable for real‐world applications.

Efforts to improve the accuracy and reliability of SERS‐based quantification have predominantly focused on the design and fabrication of nanostructured substrates. Recent innovations in substrate engineering have yielded promising results.^[^ ^8^, ^9^, ^10^, ^11^, ^12^ ^]^For instance, Yoshiki et al. developed a AuNPs/TiO₂/Au thin‐film substrate that utilized modal super coupling between LSPR and Fabry‐Perot nanocavities, achieving a 78‐fold enhancement in crystal violet detection.^[^ ^13^, ^14^ ^]^ Similarly, 3D and tunable nanostructures, such as annular cavity arrays ^[^ ^15^ ^]^ and gold nanoring arrays,^[^ ^16^ ^]^ have been shown to significantly increase hotspot density and uniformity, while innovative designs like wrinkled nano‐cone substrates fabricated on PET (Polyethylene Terephthalate) films have enabled ultra‐sensitive detection of TNT at concentrations as low as 10⁻¹^3^ mol L^−1^.^[^ ^17^ ^]^ Other approaches, such as layered double hydroxide (LDH) porous membranes on gold nanoarrays or flexible carbon cloth‐based substrates, offer scalable and cost‐effective solutions, further expanding the versatility of SERS in real‐world applications.^[^ ^18^, ^19^, ^20^, ^21^, ^22^ ^]^ In addition to advances in nanostructure fabrication, machine learning (ML) techniques have emerged as powerful tools for overcoming signal variability and enhancing quantitative accuracy.^[^ ^23^, ^24^, ^25^, ^26^, ^27^, ^28^ ^]^ ML algorithms excel at analyzing complex, high‐dimensional spectral data, identifying patterns that traditional analytical methods often fail to capture. Recent studies have demonstrated the potential of ML in SERS for tasks such as noise reduction, feature extraction, and predictive modeling.^[^ ^29^, ^30^, ^31^, ^32^, ^33^, ^34^ ^]^ For example, gradient boosting algorithms like XGBoost have achieved superior performance in quantitative predictions, while deep learning frameworks have enabled highly accurate classification of biological samples.^[^ ^35^, ^36^, ^37^ ^]^ Yang et al., for instance, developed a SERS‐based diagnostic platform incorporating ML that achieved > 99% accuracy in differentiating respiratory viruses, including SARS‐CoV‐2.^[^ ^38^ ^]^Similarly, Zhang et al. demonstrated > 97% accuracy in distinguishing breast cancer subtypes using Raman spectroscopy coupled with ML, showcasing the potential of these integrated approaches in medical diagnostics.^[^ ^39^ ^]^ Current ML methods mostly focus on processing the entire SERS spectral information to build models that improve predictive accuracy, replacing traditional peak fitting methods, or dealing with situations where SERS variation features are not pronounced. However, the inherent instability of SERS has not yet been resolved, which limits the generalization and applicability of ML models.

Despite significant advancements in SERS technology, conventional quantitative approaches relying on single‐spectrum analysis remain fraught with challenges. Single‐spectrum predictions are highly vulnerable to random disturbances, environmental noise, and inherent variability in experimental conditions. These limitations often result in inconsistent and unreliable outcomes, undermining the accuracy and reproducibility required for practical applications. A more robust alternative is leveraging multiple spectra corresponding to a single analyte concentration. This strategy captures a broader range of spectral features, mitigating the impact of signal fluctuations and enhancing predictive reliability. However, implementing this multi‐spectrum methodology effectively demands overcoming several critical challenges, including the need for diverse spectral acquisition, standardized measurement protocols, and advanced analytical methods. To address the challenge of acquiring diverse SERS spectra for a single analyte concentration, it is essential to utilize nanostructural designs capable of producing varied yet reproducible spectral responses. Fabrication strategies must ensure minimal variability while optimizing each nanostructure for consistent SERS enhancement. Additionally, parallel measurement techniques are crucial for standardizing the acquisition process. Conducting simultaneous measurements across different nanostructures under controlled conditions minimizes discrepancies caused by signal drift or environmental changes, yielding reliable data for analysis.

To advance this multi‐spectrum strategy, an approach utilizing gradient nanostructures fabricated on a single substrate is proposed in this work. Nanopatches with continuously varying areas were created in parallel using a low‐cost and efficient shadow sphere lithography (SSL) technique, eliminating variability in the fabrication process. This unique substrate design allows for simultaneous measurements of multiple feature spectra under identical experimental conditions, reducing variability during data acquisition. The gradient nanostructures produce location‐specific spectral responses, enabling the collection of diverse spectral features for a single analyte concentration. The multiple feature spectra obtained from the gradient nanostructures were analyzed using a machine learning model, making a prediction accuracy improved by 61.2% substantially higher than both traditional linear fitting methods and ML predictions based on single‐spectrum data. By leveraging this diverse spectral dataset, the ML model was able to capture subtle spectral variations, significantly enhancing the precision and robustness of concentration predictions.

## 2 Result and Discussion

### 2.1 Fabrication of Gradient Nanostructures

**Figure 1:**
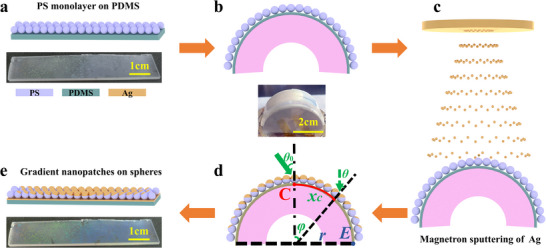
a) Formation of a PS colloidal sphere array via self‐assembly; b) Placement of the PS array on an arched block to enable gradient deposition; c) Deposition of an Ag layer onto the PS array through magnetron sputtering; d) Schematic illustration of the deposition setup, highlighting the configuration of the arched block and key parameters influencing the gradient structure; e) Final gradient Ag nanosphere shell structure on a PDMS substrate.

**Figure** **1** provides a schematic overview of the preparation process for gradient Ag nanopatches on PS spheres. The process begins with assembling hexagonally close‐packed PS colloidal monolayers (diameter, *D*) onto a PDMS (Polydimethylsiloxane) film using the water‐interface method (Figure 1a). The PDMS film is then mounted on a 3D‐printed arched block made of photopolymer resin (Figure 1b). Ag physical vapor deposition is carried out at an incident angle (*θ*_0_) on the curved PS monolayer (Figure 1c). The arched block is characterized by its curvature radius (*r*) and arc angle (*φ*), with the center (*C*) and edge (*E*) of the block marked as reference points (Figure 1d). At any position along the arc, the local angle (*φ*) and distance from the central axis (*x*_c_) are related by the equation (*x*_c_ = *r* × *φ*). Consequently, the vapor deposition angle (*θ*), relative to the central axis, varies with (*x*_c_) such that *θ* = *φ*, causing the local incidence angle to increase along the arc. In this study, *θ*_0_ = 90° and *r* = 2 cm. The mask's shadowing effect determines the position and size of nanostructures deposited on the nanosphere surface, while the gradient structure is primarily governed by the relationship *θ* (*φ*) = *x*_c_/*r*. After detaching the PDMS film from the arched block, an ordered gradient of Ag nanopatches is formed on the flexible substrate (Figure 1e). Although the PS monolayer on the flat PDMS film undergoes slight stretching when mounted onto the curved block, the inter‐center distance between neighboring nanospheres changes by less than 0.02 nm, which is negligible.^[^ ^40^ ^]^ Parameters such as *θ*_0_, *φ*, *r*, *D*, deposition thickness, and material can be systematically adjusted to control the deposition pattern, nanopatch gradient, and overall dimensions of the PDMS film and nanopatch arrays. The supplementary experiments included in Section S2 of our Supporting Information have successfully substantiated the high degree of reproducibility inherent in our sample preparation methodology.

**Figure 2:**
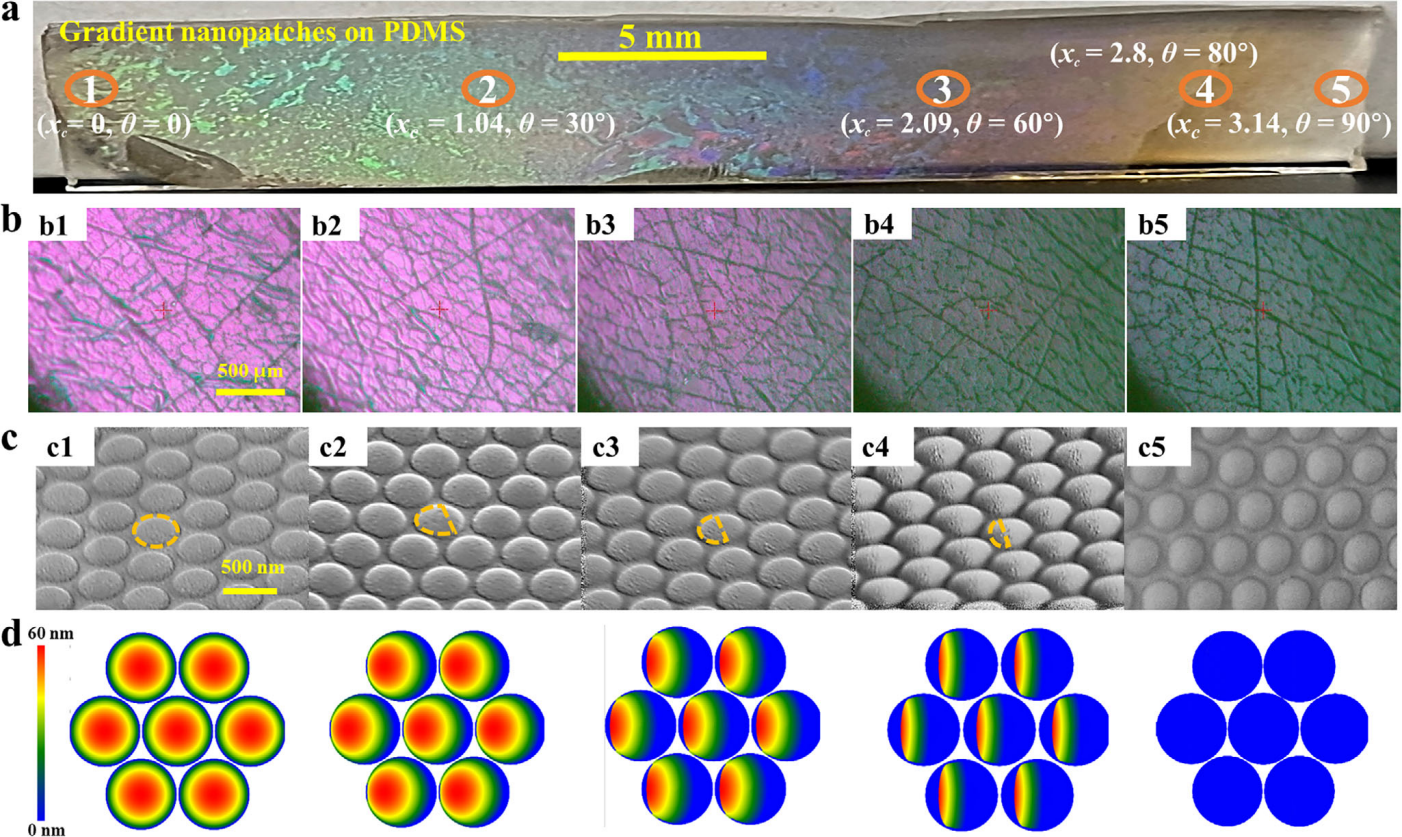
a) Photographs of the PDMS film showing gradient nanopatches, with numbered markers indicating five distinct locations along the gradient, each corresponding to specific *x*_c_ values and *θ*. b) Optical microscope images highlighting the structural variations at the five locations. c) SEM images providing detailed visualization of the nanopatch morphology at each location. d) Simulated structural models corresponding to the five locations, illustrating the gradient‐induced changes in nanopatch architecture.

**Figure** **2**a shows a macroscopic image of the PDMS sample in reflection mode, where five distinct locations along the gradient structure were analyzed: *x_c_* = 0 (*θ* = 0°), *x_c_* = 1.04 cm (*θ* = 30°), *x_c_* = 2.09 cm (*θ* = 60°), *x_c_* = 2.8 cm (*θ* = 80°), and *x_c_* = 3.14 cm (*θ* = 90°). A visible macroscopic color gradient is observed, transitioning from green (center, Location 1) to blue, pink, brown, and finally white (edge, Location 5). In contrast, control samples with arranged PS spheres but without Ag deposition exhibit no such color gradient, as their optical properties remain uniform (Figure 1a). Figure 2b presents microscope images of Locations 1 – 5 in transmission mode, showing a gradual color transition from purple‐pink to blue‐green along the gradient structure. Figure 2c provides scanning electron microscope (SEM) images of the same Locations (1 – 5). At Location 1 (*x_c_* = 0), the upper surfaces of the nanospheres are fully coated with a uniform Ag layer, forming continuous nanopatch structures. As *x_c_* increases, the Ag coverage decreases, resulting in a gradual transition from complete upper hemispherical nanopatches to partial semi‐circular shells. Figure 2d displays patterns generated by a MATLAB simulation based on calculated *θ* for Locations 1 – 5. These simulated patterns align closely with the experimental SEM observations, confirming the accuracy of the deposition gradient and the structural evolution. This correlation underscores the precise control achieved over nanopatch morphology, making the design process highly applicable for gradient nanostructured materials.

The optical color shift observed in Figure 2a,b arises from the interplay of light scattering, interference, and surface plasmon resonance (SPR), all of which are highly sensitive to the size, shape, and arrangement of nanostructures. Nanostructures with dimensions comparable to or smaller than the wavelength of light exhibit resonance scattering, selectively scattering or absorbing specific wavelengths and thereby influencing the perceived color. Moreover, the periodic arrangement of these nanostructures induces interference effects between light waves, generating structural colors.^[^ ^41^ ^]^ In gradient nanostructures, spatial variations in size, shape, and arrangement lead to position‐dependent scattering and interference, creating a continuous color gradient.^[^ ^42^ ^]^Additionally, light dispersion, where the propagation speed varies with frequency within a medium, contributes to the observed color transitions.^[^ ^43^ ^]^ The structural gradient alters the dispersion effects at different positions, further enhancing the optical gradient. Ag nanostructures introduce another critical factor: SPR. The interaction of light with free electron oscillations in the Ag layer results in selective absorption and scattering of specific wavelengths,^[^ ^44^ ^]^ amplifying the color transitions across the gradient. Together, these mechanisms synergistically contribute to the observed position‐dependent color variations in the gradient nanostructures.

### 2.2 SERS Measurement

**Figure 3:**
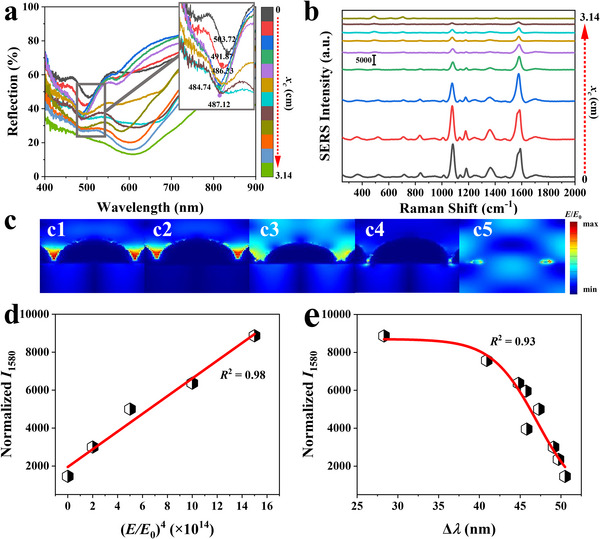
a) Reflection spectra of gradient nanopatches at different *x_c_* positions along the PDMS film. b) SERS spectra of the gradient nanopatches at various *x_c_* positions for *C*_MBA_ = 10^−4^ m. c) FDTD simulations for the gradient nanopatches at *x_c_* positions: (c1) 0 cm, (c2) 1.04 cm, (c3) 2.09 cm, (c4) 2.8 cm, and (c5) 3.14 cm. d) Normalized *I*_1580_ to nanopatch area for *C*_MBA_ = 10^−4^ m plotted as a function of (*E*/*E*_0_)^[^ ^4^ ^]^ with linear fitting. e) Function fitting of the normalized *I*_1580_ for *C*_MBA_ = 10^−4^ m versus the wavelength difference Δ*λ* between the reflection resonance dip and the excitation wavelength (532 nm).

Reflectance spectra were recorded at various locations along the gradient structure (**Figure** **3**a). At *x*_c_ = 0, a pronounced resonance dip is observed at *λ* = 584 nm, attributed to strong arising from complete Ag nanopatch coverage. As *x*_c_ increases, the resonance dip exhibits a progressive blue shift, reflecting a reduction in plasmonic coupling due to the incomplete and asymmetric Ag nanopatch structures at larger *x*_c_. The blueshift in resonance wavelength is directly correlated with the decreasing Ag nanopatch coverage (Figure 2c). At *x*_c_ = 0, the nanospheres are fully coated, forming continuous hemispherical nanopatches that enable strong coupling of localized plasmon modes. By contrast, at *x*_c_ = 1 cm, the Ag deposition produces incomplete nanopatches with reduced symmetry and coverage, weakening the plasmonic coupling and shifting the resonance to shorter wavelengths. This geometric transition disrupts the uniformity of the nanostructures, altering their interaction with incident light and generating a gradient‐dependent optical response. At *x*_c_ = 3.14, the Ag deposition is insufficient to form nanopatches, leading to the near disappearance of plasmonic coupling. Consequently, the reflectance spectrum becomes smooth, indicating a loss of resonance effects and further emphasizing the influence of structural evolution on optical properties. The SERS characteristics of the gradient samples using 4‐mercaptobenzoic acid (4‐MBA) as a Raman reporter were performed. This choice is due to the strong adsorption of MBA onto the Ag substrate through the formation of Ag‐S bonds, making the resulting SERS characteristics highly dependent on the underlying substrate structure. Figure 3b displays representative SERS spectra of MBA with the concentration *C*_MBA_ = 10^−4^ m adsorbed on the gradient nanostructures, with measurements taken from bottom to top at intervals of 0.5 cm from Locations 1 to 5 (*x_c_* = 0 to *x_c_* = 3.14 cm). Each SERS spectrum reveals two prominent peaks at 1079 and 1580 cm^−1^, which are attributed to the ring C─C stretching and C─S stretching vibrations,^[^ ^45^ ^]^ respectively. Notably, the Raman intensity of the samples decreases as the position *x_c_* increases.

The FDTD simulation was employed to model the electric field (*E*‐field) distribution of the samples at *λ* = 532 nm. The electric field depends on wavelength (Section S1, Supporting Information), while *λ* = 532 nm is the laser wavelength used in this work. Figure 3c illustrates the lateral *E*‐field distribution for Locations 1 – 5. A strong *E*‐field enhancement is observed between adjacent nanopatches for Locations 1 and 2. The *E*‐field is enhanced, which is attributed to the excitation of SPR in metal nanostructures under specific illumination wavelengths, resulting in significant local *E*‐field enhancement. The effect is particularly pronounced at tips, edges, and nanogaps of the nanostructures due to their ability to confine and concentrate the electromagnetic field effectively. The *E*‐field gradually diminishes as the position transitions from Location 1 to Location 5. This diminishing trend can be explained by the progressive change in the morphology of the Ag nanopatches along the gradient. At Location 1, the Ag nanopatches are fully formed with complete coverage. This uniform structure provides strong plasmonic coupling between adjacent nanopatches, leading to a significant enhancement of the *E*‐field. The strong coupling at nanogaps and edges effectively confines and amplifies the *E*‐field. As the position progresses toward Location 5, the Ag nanopatches coverage becomes increasingly incomplete and asymmetrical. The nanopatches transition from fully formed hemispherical structures to fragmented or almost non‐existent shells. This structural change disrupts the uniformity and reduces the interaction between adjacent nanopatches, weakening the plasmonic coupling. The incomplete nanopatches at larger *x_c_* positions (e.g., Location 4) are less effective in exciting SPR. The ability to trap and concentrate the *E*‐field diminishes because the gaps and edges—key regions for field enhancement—are no longer well‐defined or effective. As a result, the *E*‐field strength becomes progressively weaker from Location 1 to Location 5, reflecting the gradient‐dependent structural evolution of the Ag nanopatches. This transition also aligns with the observed blueshift in reflectance spectra and the loss of plasmonic resonance at larger *x*_c_.

The relationship between Raman signal intensity and the variation in *E*‐field strength was quantitatively analyzed in Figure 3d. The Raman peak intensity at 1580 cm^−1^ (*I*_1580_) for *C*_MBA_ = 10^−4^ m, normalized by the area of the Ag nanopatch structure, was linearly fitted well against the fourth power of the integrated *E*‐field (*E*/*E*_0_)^[^ ^4^ ^]^ strength with a high adjust coefficient of determination (Adj. *R* ^2^, a number that indicates the proportion of the variance in the dependent variable that is predictable from the independent variable) of 0.98, which aligns with the previous conclusion.^[^ ^45^ ^]^ Figure 3e illustrates the relationship between the normalized *I*_1580_ for *C*_MBA_ = 10^−4^ m and the wavelength difference (Δ*λ*) between the dip of the reflection spectra (representing the LSPR) and the excitation wavelength *λ* = 532 nm. The plot reveals that the normalized *I*_1580_ decreases as Δ*λ* increases, and this trend can be accurately fitted with a Logistic function, achieving a high Adj. *R* ^2^ of 0.93. This observation highlights the critical role of spectral alignment between the plasmonic resonance wavelength and the excitation wavelength in determining the efficiency of plasmonic enhancement. When the resonance wavelength closely aligns with the excitation wavelength (smaller Δ*λ*), the *E*‐field is maximized, resulting in a stronger Raman signal intensity. However, as Δ*λ* increases, the mismatch between the LSPR and the excitation wavelength leads to diminished field enhancement, thereby reducing the Raman signal intensity. This result underscores the importance of precise tuning of the nanopatch structure to optimize plasmonic coupling for applications such as SERS.

### 2.3 Machine Learning for Reliable Prediction

SERS measurements were carried out on two samples with the same manufacturing and measuring conditions. Subsequently, the two sets of data obtained from these two samples were analyzed. Fifty SERS spectra were collected for *C*_MBA_ ranging from 10^−4^ to 10^−9^ M at every location. Figure 5a shows the average *I*_1580_ and its error of the two batches for different concentrations at Location 1. For the *C*_MBA_ = 10^−4^ and 10^−5^ M, there was a significant difference between the two sets of data and large error for each batch, indicating poor data stability. As the concentration further decreases, the data stability is relatively good. Figure 5b shows the linear fitting of average *I*_1580_ with −Log (*C*_MBA_) for six locations with *x_c_* = 0.5, 1, 1.5, 2, 2.5, and 3 cm for batch 1 (solid line) and batch 2 (dashed line), with their Adj. *R* ^2^ shown in **Table** **1**. The Adj. *R* ^2^ for batch 1 and batch 2 is not stable ranging from 0.81 – 0.89 and 0.59 – 0.98, and relatively low with an average Adj. *R* ^2^ of 0.86 and 0.85, respectively, which undermines an accurate prediction. Importantly, the linear fitting for the same *x_c_* for the two batches is significantly different, which makes the prediction based on a model established based on a batch for a new measurement batch worse.

**Table 1:** Adj. *R* ^2^ of Batch 1 and 2 for different *x*_c_.

x_c_ [cm]	0.5	1	1.5	2	2.5	3
Adj. *R* ^2^ of Batch 1	0.89	0.89	0.89	0.84	0.85	0.81
Adj. *R* ^2^ of Batch 2	0.90	0.90	0.90	0.81	0.59	0.98

**Figure 4:**
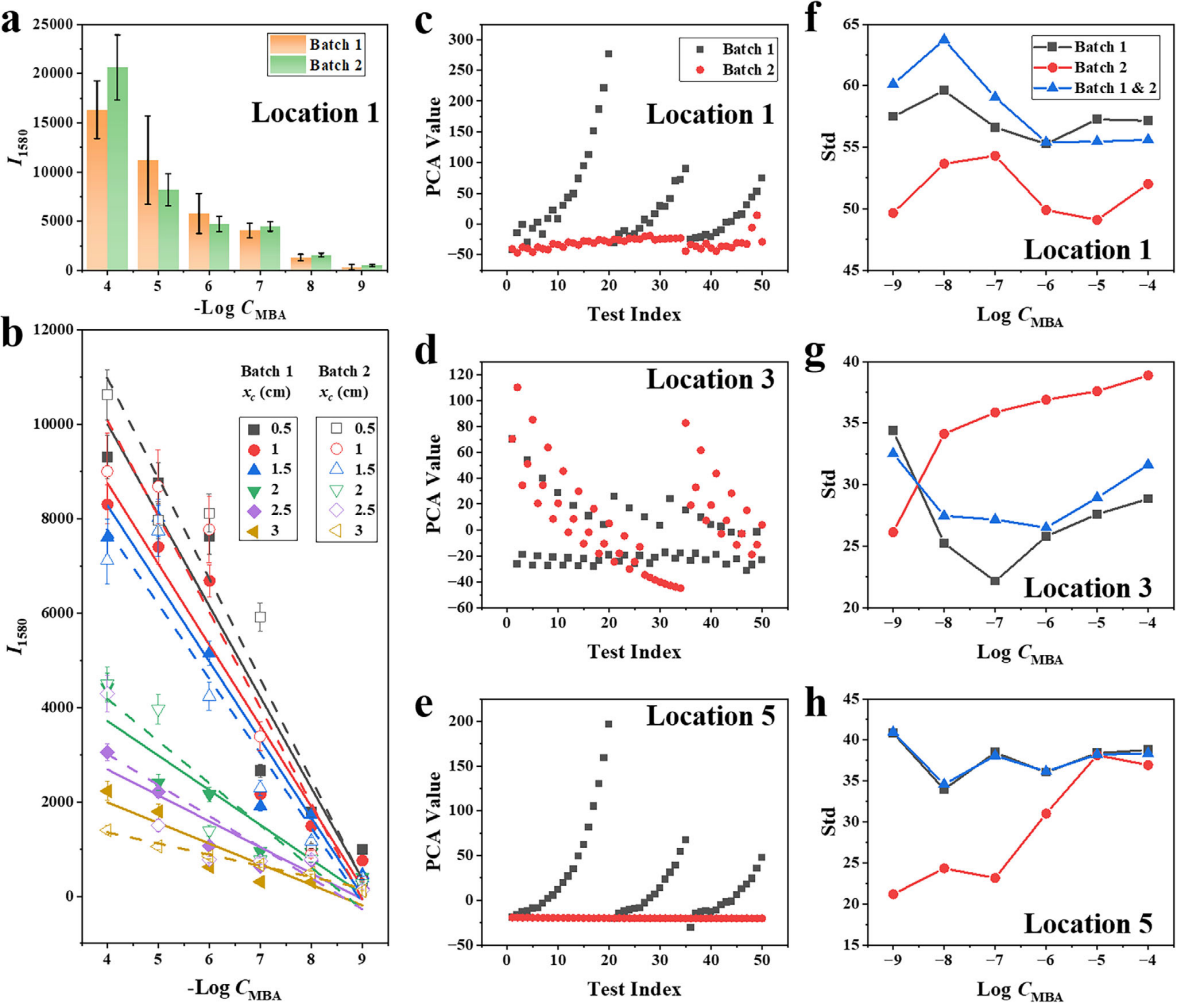
a) Histogram showing the distribution of *I*_1580_ values for Batch 1 and Batch 2 at Location 1. b) Linear fitting of the *I*_1580_ for different *x_c_* as a function of ‐Log *C*_MBA_ for Batch 1 and 2. Plots of the PCA values for the 50 tests for Batch 1 (black dots) and Batch 2 (red dots) at sample location c) 1, d) 3, and e) 5 at *C*_MBA_ = 10^−4^ m. Standard deviations of PCA‐reduced values for Batch 1, Batch 2, and combined Batch 1 & 2 at location c) 1, d) 3, and e) 5.

Principal component analysis (PCA) was utilized to transform the spectrum to one point to evaluate the stability of the whole spectrum quantitatively. PCA is a statistical procedure that employs an orthogonal transformation to convert a set of observations of possibly correlated variables into a set of linearly uncorrelated variables known as principal components. **Figure** **4**c–e illustrates the PCA‐reduced values for two batches at *C*_MBA_ = 10^−4^ m, measured at Locations 1, 3, and 5, respectively. The PCA analysis highlights both intra‐batch and inter‐batch deviations. Within Batch 1, the data shows significant variability across all positions, with larger deviations at Location 1 and slightly improved stability at higher locations. This indicates substantial inconsistency within Batch 1. Similarly, Batch 2 also exhibits some level of intra‐batch deviation, but the variability is much smaller compared to Batch 1, with PCA values tightly centered around zero at all positions. When comparing the two batches, the inter‐batch deviation is also pronounced, as Batch 1 consistently shows significantly greater variability than Batch 2. Figure 4f‐h quantitatively illustrates the standard deviations (Std) of PCA values for Batch 1, Batch 2, and Batch 1 & 2 across positions Locations 1, 3, and 5 at various *C*_MBA_. At Location 1 (Figure 4f), the combined data (Batch 1 & 2) exhibits the largest deviations, peaking at approximately 65, while Batch 2 consistently shows lower deviations, ranging from 45 to 55. The Batch 1 demonstrates intermediate variability. At Location 2 (Figure 4g), Batch 1 shows a more stable trend with deviations between 25 and 35, while Batch 2′s deviations increase progressively, peaking at ∼40. The combined batch data reflects the influence of Batch 2′s increasing variability. At Location 5 (Figure 4h), Batch 2 exhibits the lowest deviations (15–20), while Batch 1 stabilizes ≈ 35, resulting in higher combined batch variability. These results demonstrate notable intra‐batch deviations for Batch 1 and distinct inter‐batch variability, particularly at Location 1. These results suggest that not only is Batch 1 less stable internally, but there are also major differences in spectral characteristics between Batch 1 and Batch 2, indicating inconsistency both within and between the batches.

In this study, we observed significant variability in the SERS signals. This variability in SERS spectral data could be attributed to a multitude of factors, including but not limited to the roughness of the sample surface, potential damage to the sample surface from repeated laser measurements, and variations in chemical interactions. These factors can all influence the shape and intensity of the SERS spectra, thereby affecting the repeatability of the measurement results. These observations are consistent with previous research findings. To better understand these phenomena, we first reviewed the enhancement mechanisms of SERS, which primarily consist of two aspects: electromagnetic (EM) enhancement and chemical enhancement.^[^ ^46^, ^47^ ^]^ Electromagnetic enhancement mainly stems from the localized surface plasmon resonances (LSPRs) excited in metallic nanostructures, generating intense electromagnetic fields near the surface of the nanostructures.^[^ ^48^ ^]^ These so‐called “hot spots” can enhance the Raman signals by several orders of magnitude.^[^ ^49^ ^]^ Chemical enhancement, on the other hand, occurs through charge transfer interactions between the adsorbed molecules and the metal surface, which can further amplify the Raman signals, although its contribution is generally less significant compared to EM enhancement.^[^ ^50^ ^]^ The interplay and impact of these mechanisms are crucial for explaining the variability of SERS signals.

**Figure 5:**
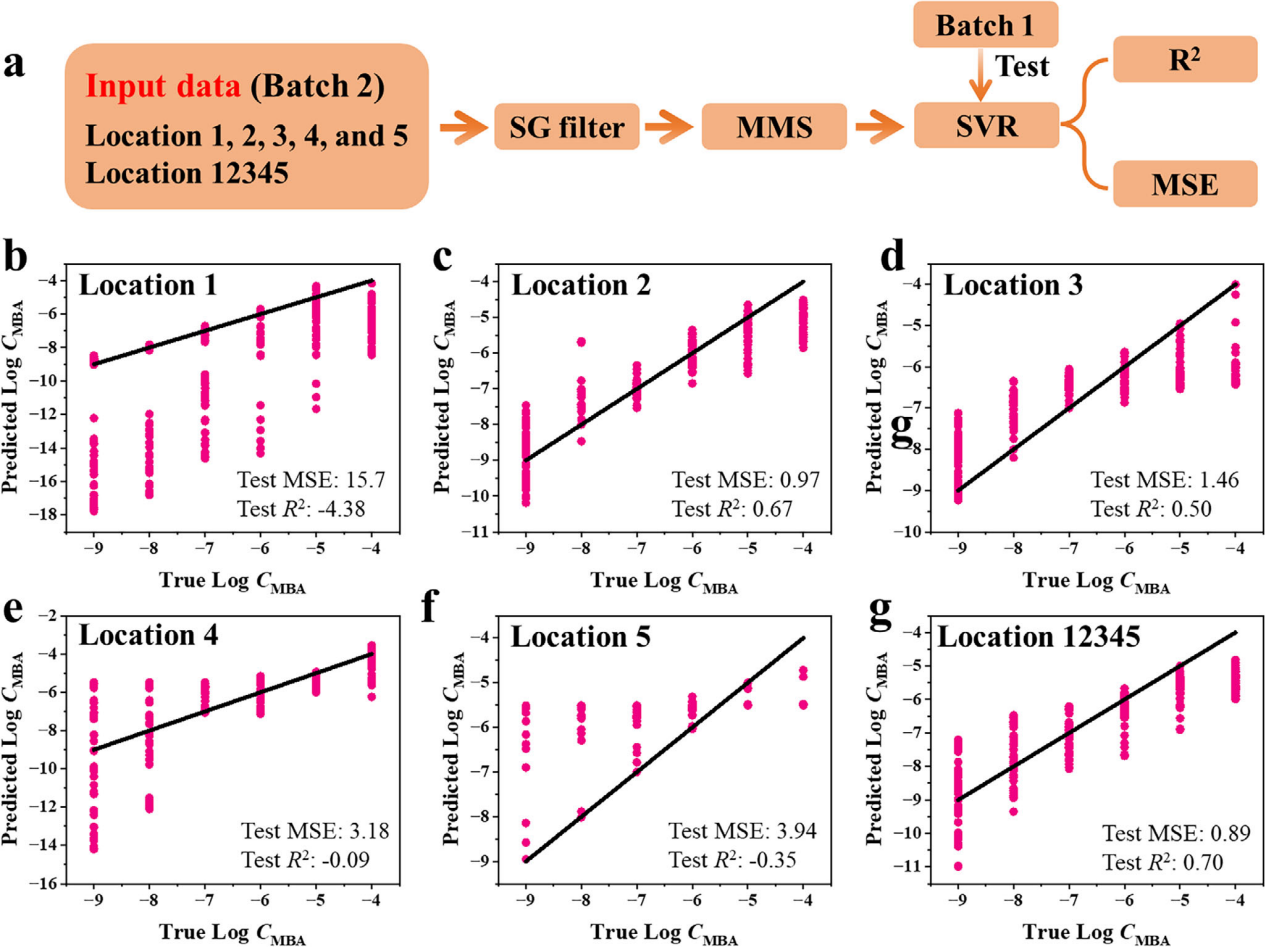
a) Workflow illustrating the process of building, training, and evaluating machine learning (ML) models for predicting *C*_MBA_. b–g) Scatter plots comparing the true *C*_MBA_ values (*x*‐axis) with the predicted *C*_MBA_ values (y‐axis) for the ML models developed at different locations: b) location 1, c) location 2, d) location 3, e) location 4, f) location 5, and g) the combined dataset from all locations (12345). The black diagonal line represents the ideal scenario where the predicted values perfectly match the true values *y* = *x*. The test MSE and *R* ^2^ are reported in the bottom‐left corner of each plot.

Due to the substantial variation observed both within a single batch and across different batches, coupled with the poor performance of linear fitting models, predictions based on linear fitting were deemed unreliable. To address the complexity and capture the underlying nonlinear relationships in the data, ML techniques were employed. The process for building and evaluating the machine learning models is illustrated in **Figure** **5**a. The dataset consists of SERS spectra with Raman shifts ranging from 350 to 2000 cm⁻¹, with *C*_MBA_ serving as the target labels. Data from Batch 2 was used for training, with a 7: 3 splits between the training and validation sets. The model development begins with the preprocessing of input data, which includes spectra from individual Locations (1 to 5) as well as a combined dataset (Locations 12345). The raw spectra are first smoothed using a Savitzky‐Golay (SG) filter to reduce noise and subsequently normalized using Min‐Max Scaling (MMS).^[^ ^51^ ^]^ The processed data is then fed into a support vector regression (SVR) ^[^ ^52^ ^]^ model for training. The performance of the trained model is evaluated on data from Batch 1, which serves as the test set. Two key metrics are used to assess predictive performance: the coefficient of determination (*R* ^2^)^[^ ^53^ ^]^ and the mean squared error (MSE).^[^ ^54^ ^]^ The (*R* ^2^) value quantifies how well the model explains the variance in the data, with values closer to 1 indicating better performance, while the MSE measures the average squared differences between predicted and actual values, with smaller values reflecting higher accuracy.

Figure 5b–g shows the comparison of true versus predicted values for models trained on individual locations (Figure 5b–f) and the combined dataset (Figure 5g). The black diagonal line in each scatter plot represents the ideal scenario where predicted values perfectly match true values, and the test MSE and *R* ^2^ values in the bottom‐left corner quantify the predictive performance of each model. For Location 1 (Figure 5b), the model demonstrates poor predictive performance, with a high test MSE of 15.7 and a low *R* ^2^ of 0.38. The scatter plot shows significant dispersion of points around the diagonal line, indicating that the model struggles to generalize well. This could be due to the instability of the data across different experimental batches, resulting in inconsistent patterns that are hard for the model to capture. Similarly, for Location 3 (Figure 5d), the model exhibits moderate performance, with a test MSE of 1.46 and an *R* ^2^ of 0.50. While some alignment with the diagonal line is observed, the predictions still show noticeable variation, likely caused by batch‐to‐batch inconsistencies. Among the individual location models, Location 2 (Figure 5c) achieves the best results, with a test MSE of 0.97 and an *R* ^2^ of 0.67. The scatter plot shows a tighter clustering of points near the diagonal line, suggesting that the data for this location may be more stable across batches, allowing the model to identify clearer predictive patterns. However, Locations 4 (Figure 5e) and 5 (Figure 5f) perform poorly, with test MSEs of 3.18 and 3.94, and negative *R* ^2^ values of −0.09 and −0.35, respectively. These results indicate that the models for these locations fail to capture meaningful relationships, likely due to severe data instability or inconsistencies between different experimental batches. The wide scatter of points in the respective plots reflects this lack of predictive accuracy. we observed that the machine learning model performed less effectively at Location 1 compared to the overall dataset. This may be due to higher noise levels or significant baseline drift in the spectral data at this location, which affects the consistency of the data. Additionally, Location 1 might have unique electromagnetic enhancement effects or distinct chemical environments, leading to greater variability in spectral features.

In the field of Surface‐Enhanced Raman Scattering (SERS) spectral analysis, data variability is indeed a significant issue, caused by a multitude of factors. Variations in spectral shape may arise from spectral modulation due to secondary electromagnetic enhancement effects, and alterations in chemical interactions can also lead to changes in SERS spectra.^[^ ^55^, ^56^ ^]^ Furthermore, discrepancies in SERS spectral data at the same location may result from damage to the sample surface caused by multiple laser measurements.^[^ ^57^ ^]^ The interplay of these numerous factors makes it challenging to precisely identify and distinguish the specific causes of variation in practical applications, which is the root of the reproducibility issues in SERS.^[^ ^58^ ^]^ Recognizing this, understanding and suppressing these variations are crucial for enhancing the accuracy and reliability of SERS technology. Machine learning (ML), as a sophisticated data analysis tool, has shown great potential in dealing with such complex data variability. The key advantage of ML methods is that they focus on processing SERS spectral data without the need to specifically identify the causes of variation. By employing algorithms, ML can learn from the data and recognize key patterns that affect prediction accuracy, thereby enabling precise analysis and forecasting of SERS spectral data. This approach offers a novel perspective for the application of SERS technology, especially in the face of complex data variability, providing an effective solution to enhance the reproducibility and reliability of SERS measurements.

In line with this approach, our study leverages ML to address the instability of SERS signals. The model trained on the combined dataset (Locations 12345) (Figure 5g) achieved the best overall performance, with a test MSE of 0.89 and an *R* ^2^ of 0.70. The scatter plot shows a strong alignment of points along the diagonal line, demonstrating that pooling data from all locations significantly enhances the model's generalization ability. When compared to the average test MSE (5.85) and *R* ^2^ (0.22) of the individual location models (Figure 5b–f), the test MSE of the combined dataset model was reduced by 84.8%, reflecting a substantial improvement in predictive accuracy. Furthermore, the *R* ^2^ improved by 61.2%, indicating that the combined dataset model explained a much larger proportion of the variability in the target variable relative to the average performance of the single‐location models. This improvement can be attributed to the integration of diverse data from multiple locations, which exposes the model to a broader range of patterns and reduces the influence of batch‐to‐batch variability that hindered the individual models. By leveraging the complementary information across locations, the combined dataset approach enhances robustness and enables the model to better capture the underlying trends, underscoring the value of dataset integration in addressing data instability and improving overall predictive performance. To evaluate the predictive and analytical capabilities of the machine learning model across a variety of analytes, we conducted an in‐depth investigation into the Surface‐Enhanced Raman Scattering (SERS) characteristics of gradient samples using 4‐aminothiophenol (4‐ATP) as a Raman probe molecule. The detailed results, provided in Sections S4 and S5 (Supporting Information), collectively demonstrate that the machine learning model also possesses exceptional analytical prowess, enabling it to predict the behavior of additional analytes effectively.

## 3 Conclusion

This study addresses the critical challenges of variability and inconsistency in SERS quantification by integrating gradient nanostructure design with ML. Gradient Ag nanopatches were fabricated on flexible PDMS substrates using shadow sphere lithography (SSL), enabling the generation of reproducible, location‐specific spectral responses. The controlled evolution of nanopatch morphology along the gradient was characterized, revealing position‐dependent plasmonic coupling that influenced optical and SERS properties. A multi‐spectrum analysis strategy utilizing the spectral diversity from these gradient nanostructures was implemented with ML algorithms. The combined dataset, integrating data from all gradient locations, achieved significant improvements in predictive performance, reducing the test MSE by 84.8% and increasing the *R* ^2^ by 61.2% compared to single‐location models. This approach mitigated batch‐to‐batch variability, captured subtle spectral variations, and enhanced the robustness and accuracy of analyte concentration predictions. By combining gradient nanostructures with advanced data‐driven models, this work provides a scalable and cost‐efficient framework for reliable quantitative SERS analysis. The findings represent a significant step toward transitioning SERS into a robust tool for real‐world applications, including chemical sensing, biological diagnostics, and environmental monitoring, advancing its capabilities beyond traditional qualitative methods.

## 4 Experimental Section

### Fabrication of Gradient Nanostructures

First, a PDMS (Polydimethylsiloxane, thickness 2 mm, Hefei Ke Liao New Material Technology Co., Ltd) film was washed with ethanol (Shanghai Aladdin Biochemical Technology Co., Ltd, 99%) and Ultrapure water (18.25 mΩ·cm) serially. After dried at room temperature, the PDMS film was treated for 100 s by Plasma (PT‐5S) cleaning at a power of 100 W to get the neat and hydrophilic surface, which was divided into rectangular of 2.0 × 6.4 cm. Then, the gas‐liquid interface self‐assembly technique was used to arrange a 2D ordered polystyrene (PS) (An aqueous solution of 10 wt % monodisperse PS colloidal particles with an average diameter of 500 nm and a density of 1.05 g cm^−3^ was purchased from TSSphere Co., Ltd, China) colloidal sphere template array on the PDMS. The PS colloidal spheres (size of 500 nm) were assembled into a hexagonal packed array on PDMS film. Next, the PDMS film with PS colloidal spheres arranged was installed on an arched block made of photopolymer resin for a 3D printer. Finally, the PDMS surface on arch block were coated with Ag film by magnetron sputtering system at a rate of 1.0 nm s^−1^ in vacuum chamber (vacuum of 4.5 × 10^−4^ Pa). Ag [Target materials (Ag 99.99%), Beijing TIANRY Science & Technology Developing Center.] was sputtered with a working power of 10 W, an Ar gas flow of 25 sccm, and a working pressure of 1.0 Pa. A 60 nm thick Ag layer was deposited on the substrate.

### SERS Measurement

Confocal Raman spectroscopy (XperRAM‐S700), equipped with a 532 nm laser, was used for Raman measurements. A 50× microscope objective was employed to focus the laser onto the sample surface with a laser power of 50 mW. The exposure time for one mapping pixel was 3 seconds, with a spectral range of Raman shift between 250 and 2000 cm^−1^. The 4‐MBA (4‐Mercaptophenzoic acid, Shanghai Aladdin Biochemical Technology Co., Ltd, 99%) was dissolved in absolute ethanol (Shanghai Aladdin Biochemical Technology Co., Ltd, 99%) to prepare solutions of varying concentrations. Samples were immersed in a 10 mL ethanol solution of 4‐MBA for 30 min, after which they were washed for 5 min in absolute ethanol to remove unabsorbed molecules, and then dried at room temperature. Similarly, 4‐Aminothiophenol (4‐ATP), also at 99% purity from Shanghai Aladdin Biochemical Technology Co., Ltd., was dissolved in absolute ethanol to prepare solutions of different concentrations. Samples were immersed in a 10 mL ethanolic solution of 4‐ATP for 30 min. Subsequently, they were washed for 5 min in absolute ethanol to eliminate unabsorbed molecules, and then air‐dried at ambient temperature.

### FDTD Calculation

Simulations of the nanopatch structures, varying with deposition angle, were performed using MATLAB (MathWorks Inc.) to model their formation and subsequently importing the structures into the finite difference time domain (FDTD) method (FDTD Solutions, Lumerical Solutions Inc.) to calculate the electromagnetic (EM) field distribution. The simulations were processed sequentially, with each simulation commencing only after the previous one was completed. The smallest periodic unit of the array structure was used as the simulation domain, allowing for position‐specific modeling of the nanostructures across the sample. A plane wave with a wavelength of 532 nm was employed as the light source to evaluate the influence of structural spacing on the EM field distribution in the *x*‐*y* plane. The FDTD simulation grid resolution was set to 7 for precise calculations. Materials included in the simulations were selected from the software's material database and comprised PDMS, PS, and Ag (Ag, 0 – 2 µm). This simulation approach enabled accurate analysis of the electromagnetic behavior of the gradient nanostructures, providing key insights into their plasmonic properties.

### Characterization

Ultrahigh‐resolution field‐emission SEM (JOEL/JSM‐IT500HR) was used to evaluate morphologies. The absorption spectra of nanostructures were measured using a confocal micro‐area Raman scattering instrument (K‐Sens). Additionally, the Ag film situation was also simulated at the corresponding position of the sample using MATLAB (MathWorks Inc.).

## Conflict of Interest

The authors declare no conflict of interest.

## Supporting information



Supporting Information

## Data Availability

The data that support the findings of this study are available from the corresponding author upon reasonable request.
