# Supplementary material for: Gradient Nanostructures and Machine Learning Synergy for Robust Quantitative Surface‐Enhanced Raman Scattering
Source: Adv Sci (Weinh). 2025 Apr 25;12(26):2501793. doi: 10.1002/advs.202501793 (PMC12244994; doi:10.1002/advs.202501793)
Supplement: Supplementary file 1 — Supporting Information [file ADVS-12-2501793-s001.docx]

Supporting Information

**Gradient Nanostructures and Machine Learning Synergy for Robust Quantitative Surface-Enhanced Raman Scattering**

*Xiaoyu Zhao^1^*^§^*, Yuxia Wang^1^*^§^*, Yuting Liu^2^*^§^*, Xinyi Chen^2^, Mingyu Cheng^2^, Yaxin Wang^1^, Jiahong Wen^3^, Renxian Gao^1^, Kun Zhang^1^, Fengyi Zhang^1^, Rufei Cui^1^, Yongjun Zhang^1*^, Zengyao Wang^4*^, and Bin Ai^2*^.*

^1^College of Materials and Environmental Engineering, Hangzhou Dianzi University, Hangzhou, Zhejiang 310018, P. R. China

^2^School of Microelectronics and Communication Engieerimng, Chongqing Key Laboratory of Bio-perception & Intelligent Information Processing, Chongqing University, Chongqing, P RChina 400044

^3^The College of Electronics and Information, Hangzhou Dianzi University, Hangzhou 310018, China; Shangyu Institute of Science and Engineering, Hangzhou Dianzi University, Shaoxing, Zhejiang 312000, China

^4^Shandong Second Medical University, Weifang, 261053 Shandong, PR Shandong, China.

Corresponding Author’s Email: [yjzhang@hdu.edu.cn](mailto:yjzhang@hdu.edu.cn); [zengyao@sdsmu.edu.cn](mailto:zengyao@sdsmu.edu.cn); binai@cqu.edu.cn

§Xiaoyu Zhao, Yuxia Wang and Yuting Liu and contributed equally to this paper

**Section S1. Wavelength-Dependent Electric Field Strength and Reflectance Spectra Analysis via FDTD Simulation**


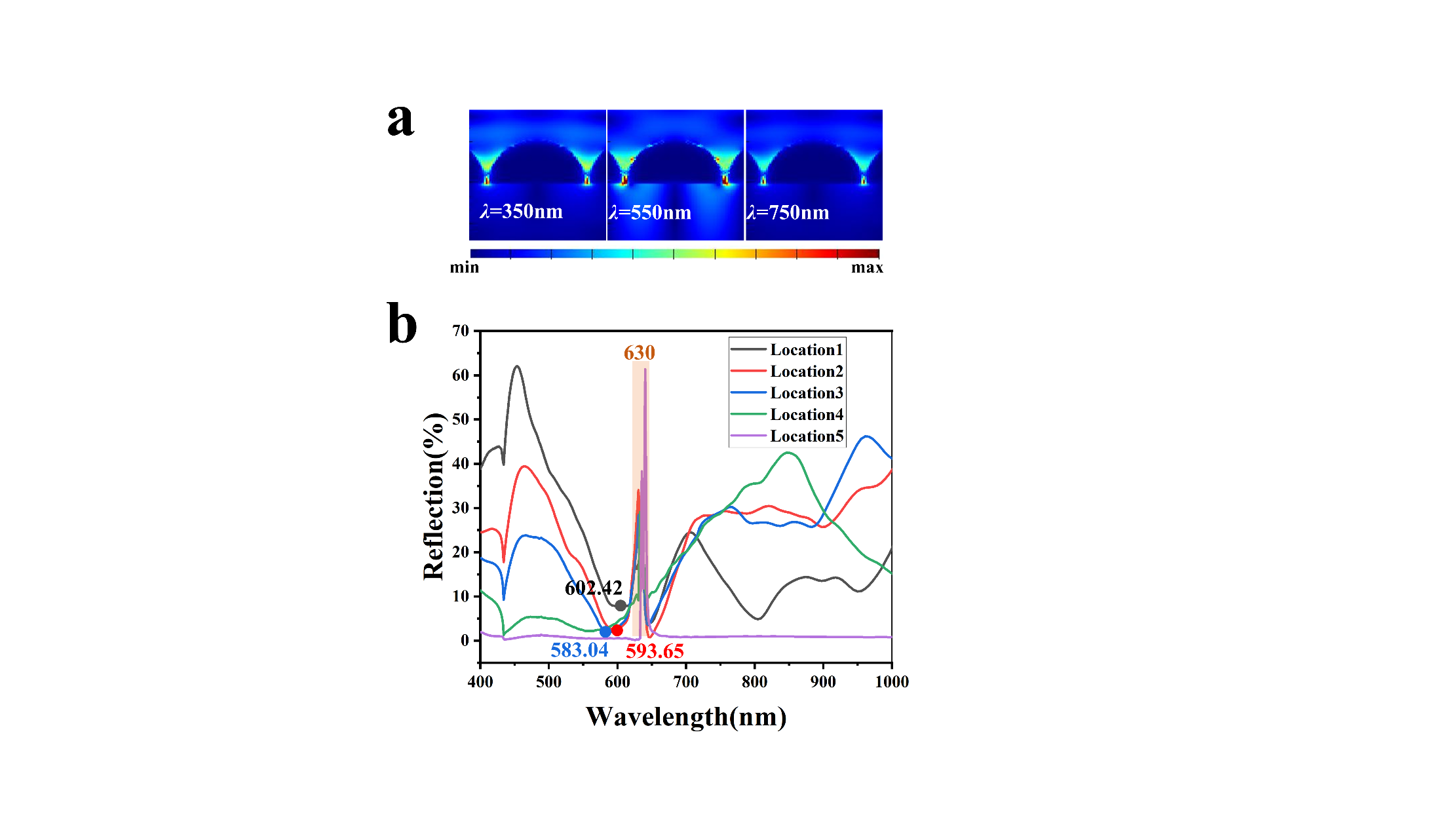


**Figure S1**. (a) The wavelength dependence of the electric field strength calculated by the FDTD method; (b) The reflectance spectra of the sample at different positions calculated by FDTD.

we utilized FDTD method to simulate the distribution of the electric field intensity of the samples at different wavelengths to explore their wavelength dependence. **Figure S1a** illustrates the electric field distribution of the samples at three specific wavelengths (350nm, 550nm, and 750nm), The calculated electric field intensities within the same range at various wavelengths are 4824, 6216, and 5714, respectively. These simulation results provide us with the optical response characteristics of the samples under different illumination conditions. From **Figure S1a**, it can be observed that the distribution pattern of the electric field changes significantly with the increase in wavelength. At the shorter wavelength (350nm), the electric field is primarily concentrated on the surface of the sample and is relatively uniform. This indicates that at shorter wavelengths, the sample's response to light is mainly characterized by surface effects, with weaker enhancement effects of the electric field intensity within the sample. As the wavelength increases to 550nm, the electric field distribution begins to show local enhancement, especially in certain specific areas of the sample. This phenomenon can be attributed to the Surface Plasmon Resonance (SPR) effect, where in metallic nanostructures, when the wavelength of the incident light matches the resonant wavelength of the surface plasmon, a strong enhancement of the electric field is produced on the surface of these structures. This enhancement effect is particularly evident at the tips, edges, and nanogaps of the nanostructures, as these areas can effectively confine and concentrate the electromagnetic field. When the wavelength further increases to 750nm, the regions of electric field enhancement become more extensive, and the intensity is somewhat reduced. This may be due to the increased penetration depth of the incident light at longer wavelengths, leading to a more uniform distribution of the electric field within the sample, thereby reducing the local enhancement effect. Additionally, the SPR effect at longer wavelengths may not be as significant as at shorter wavelengths, as the resonance conditions for SPR are highly sensitive to wavelength. In summary, the simulation results of the electric field intensity's wavelength dependence shown in **Figure S1a** indicate that the optical response characteristics of the samples change significantly with the variation in the wavelength of the incident light. These changes are mainly reflected in the distribution pattern and enhancement effect of the electric field, providing us with important clues for understanding the optical behavior of the samples. The simulated reflection spectrum in Figure S1b shows a significant decrease in reflectivity at Location1 at 602.42nm, indicating the presence of a resonance peak. This is consistent with the enhanced electric field intensity at 550nm wavelength observed in Figure S1a, confirming the correlation between enhanced electric fields and reduced reflectivity. The localized enhancement of the electric field at 550nm, particularly at the tips, edges, and nanogaps of the nanostructures, is due to the Surface Plasmon Resonance (SPR) effect. This is interconnected with the decrease in reflectivity at 602.42nm wavelength, reflecting the optical characteristics of the nanostructures at specific wavelengths. In summary, the simulation results from Figures S1a and S1b reveal the changes in electric field distribution and reflectivity of nanostructures at different wavelengths, providing crucial clues for understanding their optical behavior and offering guidance for the design and optimization of nanophotonic devices.

We employed the Finite Difference Time Domain (FDTD) method to simulate the reflection spectrum of the optical sample and compared the simulation results with the experimental measurements (**Figure S1b**). Both the simulation results and the experimental data show a blue shift in the peaks and valleys of the reflection spectra formed in the visible range as the testing position of the sample changes from position 1 to position 5, and an overall decrease in reflectance. This indicates that both methods have captured the fundamental optical behavior of the sample. Additionally, both the simulation and experimental results exhibit a broad reflection band in the visible region, which is consistent with the expected response of the sample material. Despite the overall consistency between the simulation results and experimental data, there are still notable discrepancies between the two. Specifically, the peak valleys observed at around 500 nm in the experiment and at around 600 nm in the simulation can be attributed to several factors. First, the actual size distribution of the microspheres deviates from the idealized model used in the simulation. Variations in the diameter of the microspheres can lead to shifts in the resonance wavelengths. Second, the refractive index settings in the simulation may not fully match the actual refractive index of the polystyrene microspheres or the surrounding medium. This mismatch can cause shifts in the resonance wavelengths and affect the intensity of the peaks. Additionally, measurement errors during the experimental process or limitations in the detection system may further contribute to the deviations in peak positions between the experiment and simulation. Moreover, the simulated reflection spectrum exhibits a distinct peak at 630 nm, which was not observed in our experimental measurements. This discrepancy can be explained by the surface roughness and structural inhomogeneity of the microspheres in the experiment, which deviate from the ideal conditions assumed in the simulation. These imperfections can broaden or shift the resonance peaks, making it difficult to capture the sharp feature peak at 630 nm as seen in the simulation.

**Section S2. Spectral Reflectance Comparison of Different Sample Batches**
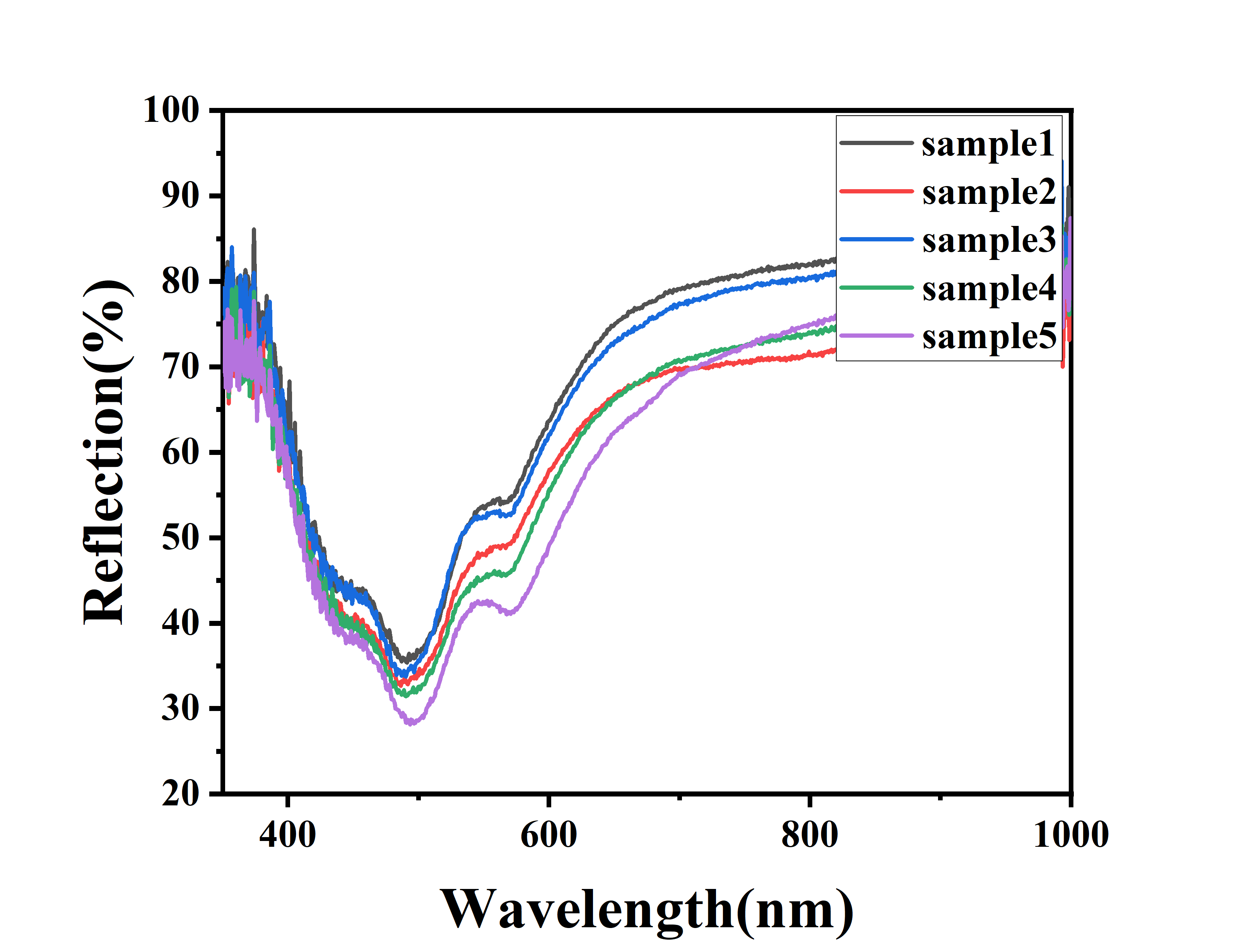


**Figure S2.** Comparison of Spectral Reflectance at the Same Location for Different Batches of Samples (Sample1 to Sample5) under Identical Experimental Conditions.

We have provided further supplementation to more comprehensively demonstrate the repeatability of our experiments. We have included reflection spectra data for different batches of samples at the identical location under strictly consistent experimental conditions (**Figure S2**). These spectral data not only substantiate the high degree of repeatability of our fabrication method but also offer quantitative evidence for evaluating the consistency of the sample performance. Specifically, we conducted detailed spectral reflectance tests on five batches of samples independently prepared (designated as sample1 through sample5). Each batch was assessed under the same measurement conditions to ensure the comparability of the test results. By comparing the reflection spectra of these samples across the wavelength range from 400 nm to 1000 nm, we observed a high level of consistency in the overall trends and key characteristics of their reflectance curves. The consistency of these spectral data indicates that our fabrication process is not only capable of repeatedly producing gradient nanostructures with the expected optical properties but also that the performance of these structures is highly stable across different batches. This high level of repeatability and stability is crucial for the further application and development of the material, as it ensures the reliable large-scale production of products with consistent performance.

**Section S3. Gradient Nanopatches SERS Spectra for *C*_ATP_ = 10⁻⁴ M at Different Positions**


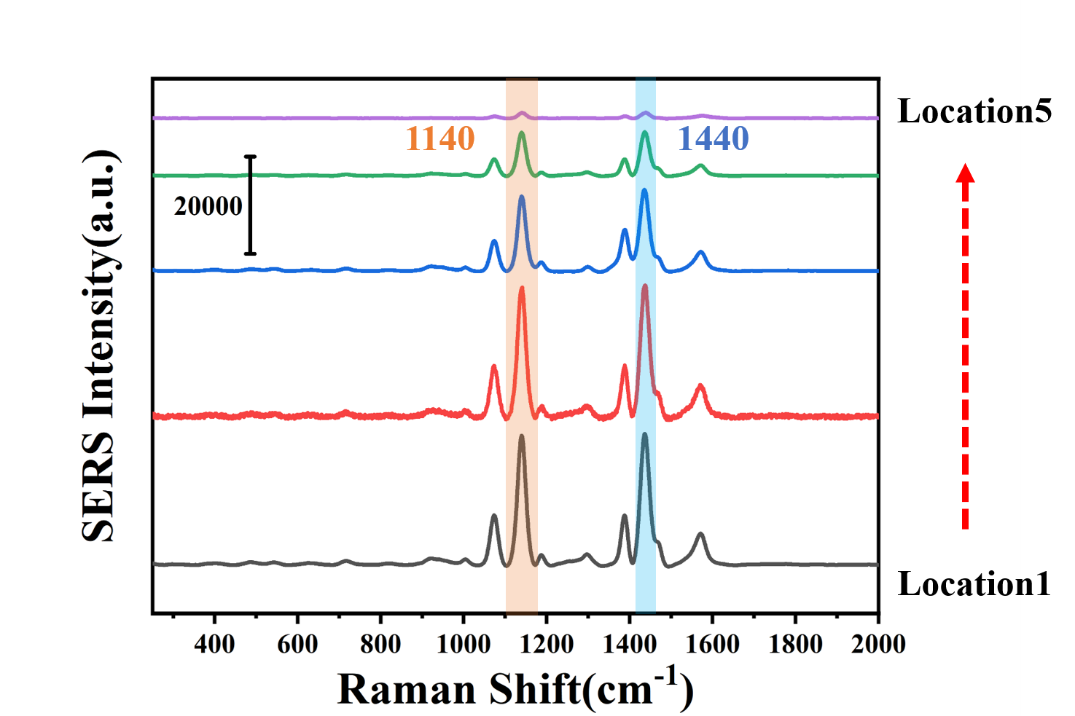


**Figure S3.** SERS spectra of the gradient nanopatches at various *x_c_* positions for *C*_ATP_ = 10^-4^ M.

4-Aminothiophenol (4-ATP) was used as a Raman reporter molecule to investigate the SERS characteristics of gradient samples. 4-ATP can strongly adsorb onto the surface of silver nanoparticles through its thiol group (-SH), thereby generating significant SERS signals. **Figure S3** shows the representative SERS spectra of 4-ATP adsorbed on gradient nanostructures at a concentration of C_ATP_ = 10⁻⁴ M, measured at locations 1 to 5 (*x_c_* = 0 to *x_c_* = 3.14 cm). Two prominent characteristic peaks are observed in each SERS spectrum, located at 1140 cm⁻¹ and 1440 cm⁻¹, which are attributed to the C-H bending vibration and the C=C stretching vibration of the benzene ring, respectively. Notably, the Raman intensity of the samples gradually decreases with increasing position.

**Section S4. Comparison of True vs. Predicted *C*_ATP_ Values for ML Models at Different Locations and Combined Dataset**
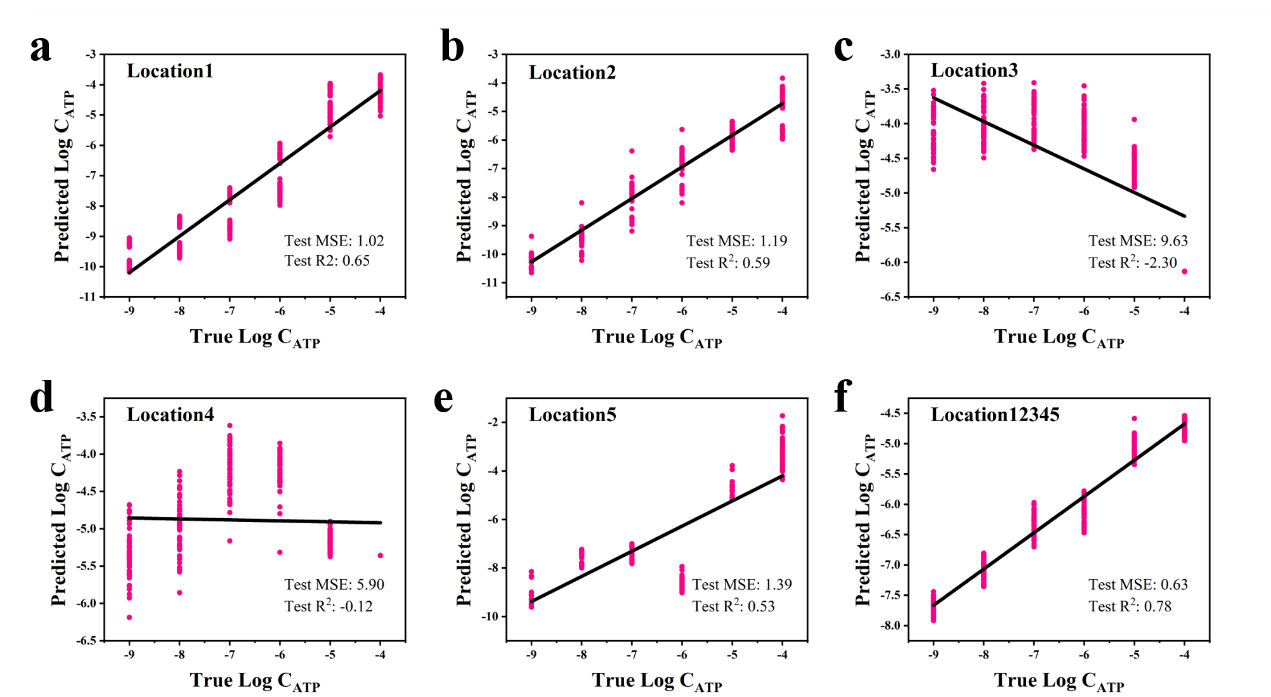


**Figure S4.** (a-f) Scatter plots comparing the true *C*_ATP_ values (*x*-axis) with the predicted *C*_ATP_ values (y-axis) for the ML models developed at different locations: (b) location 1, (c) location 2, (d) location 3, (e) location 4, (f) location 5, and (g) the combined dataset from all locations (12345). The black diagonal line represents the ideal scenario where the predicted values perfectly match the true values *y* = *x*. The test MSE and *R*^2^ are reported in the bottom-left corner of each plot.

**Figure S4a-f** shows the comparison between true and predicted values for models trained at individual locations (**Figure S4a-e**) and the combined dataset (**Figure S4f**). The black diagonal line in each scatter plot represents the ideal scenario where predicted values perfectly match the true values, while the test mean squared error (MSE) and coefficient of determination (R²) values in the bottom-left corner quantify the predictive performance of each model. For locations 3 (**Figure S4c**) and 4 (**Figure S4d**), the models exhibit poor predictive performance, with test MSE values as high as 9.63 and 5.90, respectively, and R² values as low as -2.30 and -0.12. The scatter plots show significant dispersion of data points around the diagonal line, indicating that the models struggle to generalize. This may be due to the instability of data across different experimental batches, making it difficult for the models to capture consistent patterns. Similarly, for locations 1 (**Figure S4a**), 2 (**Figure S4b**), and 5 (**Figure S4e**), the models show moderate performance, with test MSE values around 1 and R² values ranging from 0.53 to 0.65. Although some alignment with the diagonal line is observed, the predictions still exhibit noticeable fluctuations, likely due to batch-to-batch inconsistencies. In contrast, the model trained on the combined dataset (locations 12345) (**Figure S4f**) achieves the best overall performance, with a test MSE of 0.63 and an R² value of 0.78. The scatter plot shows strong alignment of data points along the diagonal line, indicating that integrating data from all locations significantly enhances the model's generalization ability. Compared to the average test MSE (3.83) and R² (-0.31) of the individual location models (**Figure S4a-e**), the combined dataset model reduces the test MSE by 83.6%, reflecting a significant improvement in predictive accuracy. Additionally, the R² value increases by 41.1%, suggesting that the ML model also has the same predictive analytical capability for other analytes.
